# Targeting Tumor Differentiation Grade-related Genes Prognostic Signature Including COL5A1 Based on Single-cell RNA-seq in Gastric Cancer

**DOI:** 10.7150/ijms.107509

**Published:** 2025-05-07

**Authors:** Jianming Wei, Xibo Gao, Zhufeng Li, Yangpu Jia, Chuan Li, Jian Liu

**Affiliations:** 1 Department of General Surgery, Tianjin Medical University General Hospital, Tianjin, China; 2 Department of Dermatology, Tianjin Children's Hospital, Tianjin, China

**Keywords:** Gastric cancer, tumor differentiation grade, single-cell RNA sequencing, Cox regression, B cells memory

## Abstract

**Background:** Tumor differentiation grade was reported to be a prognostic factor in gastric cancer (GC). Here, we identify a novel tumor differentiation grade-related genes prognostic signature and provide new biomarkers using single-cell RNA sequencing (scRNA-seq) in GC.

**Methods:** ScRNA-seq profiles of GC were analyzed by 'seurat' package. Tumor differentiation grade module was identified through a weighted gene co-expression network analysis (WGCNA). Hematoxylin and eosin (H&E) were performed to classify differentiation grade. The effects of tumor differentiation grade on prognosis were explored using the Kaplan-Meier. Tumor differentiation grade prognostic signature was constructed and validated in GC.

**Results:** Using GEO database, the scRNA-seq cell differentiation, clusters, and marker genes were identified in GC. Functional enrichment analysis revealed that common differentially expressed genes (DEGs) were mainly enriched in neutrophil process. Integrating clinical factors in GC, WGCNA analysis indicated that tumor differentiation grade module was the most significant. H&E and Kaplan-Meier analysis revealed that well-differentiated had a better prognosis in GC. Subsequently, tumor differentiation grade-related genes signature was established and validated (TNFAIP2, MAGEA3, CXCR4, COL1A1, FN1, VCAN, PXDN, COL5A1, MUC13 and RGS2). Cox regression analysis showed that age, TNM stage and the risk score were significantly associated with prognosis. And then, these genes could predict prognosis in GC. Finally, the hub gene COL5A1 was obviously correlated with B cells memory, dendritic cells activated, macrophages M0, macrophages M2, plasma cells, T cells follicular helper in GC.

**Conclusions:** This study reveals a novel tumor differentiation grade-related genes signature, and COL5A1 represents a promising biomarker in GC.

## Introduction

Gastric cancer (GC) is an aggressive tumor ranking fourth for mortality globally^1^, and its incidence rate is increasing over the recent decades. Although treatments with surgery and chemotherapy lead to a satisfactory survival benefit^2^, ^3^, the 5-year overall survival rate of GC is still low. Increasing biomarkers such as circular RNAs, microRNAs, and tumor microenvironment‑related genes have been identified to predict prognosis in GC^4^-^6^. Therefore, it is important to excavate the predictive biomarkers and find a novel strategy for GC.

With the increasing studies of cancer pathology, the tumor differentiation grade has been shown to be a prognostic factor in many cancers. Kristoffer derwinger et al. reported that the tumor differentiation grade was correlated significantly with the overall TNM stage and the risk of having lymph node metastasis^7^. A previous study had shown that apoptosis and cell proliferation correlated with tumor differentiation grade in patients with lung adenocarcinoma^8^. However, the prognostic risk model of tumor differentiation grade-related genes in GC was unexplored.

Single-cell RNA sequencing (scRNA-seq) is applied to explore the cellular components and gene expression at the single-cell level^9^, and can provide a new insight into tumor heterogeneity. Moreover, several studies have elucidated the association between TME and intra-tumoral heterogeneity using scRNA-seq^10^, ^11^. ScRNA-seq is also a powerful way for identifying anti-cancer drug response^12^, ^13^. However, the potential roles of the tumor differentiation grade-related genes and prognostic signature based on scRNA-seq have not been elucidated yet.

The present study develops a novel tumor differentiation grade-related genes prognostic signature which include ten genes (TNFAIP2, MAGEA3, CXCR4, COL1A1, FN1, VCAN, PXDN, COL5A1, MUC13 and RGS2). These genes are also highly connected in the protein-protein interaction (PPI) network. The hub gene COL5A1 is supposed to be significantly associated with tumor infiltrating immune cells and can be potential targets for prognosis in GC. Our data could support a deeper understanding of tumor differentiation grade-related genes in GC to provide appropriate therapy strategies.

## Materials and methods

### The study flowchart

**Figure 1:**
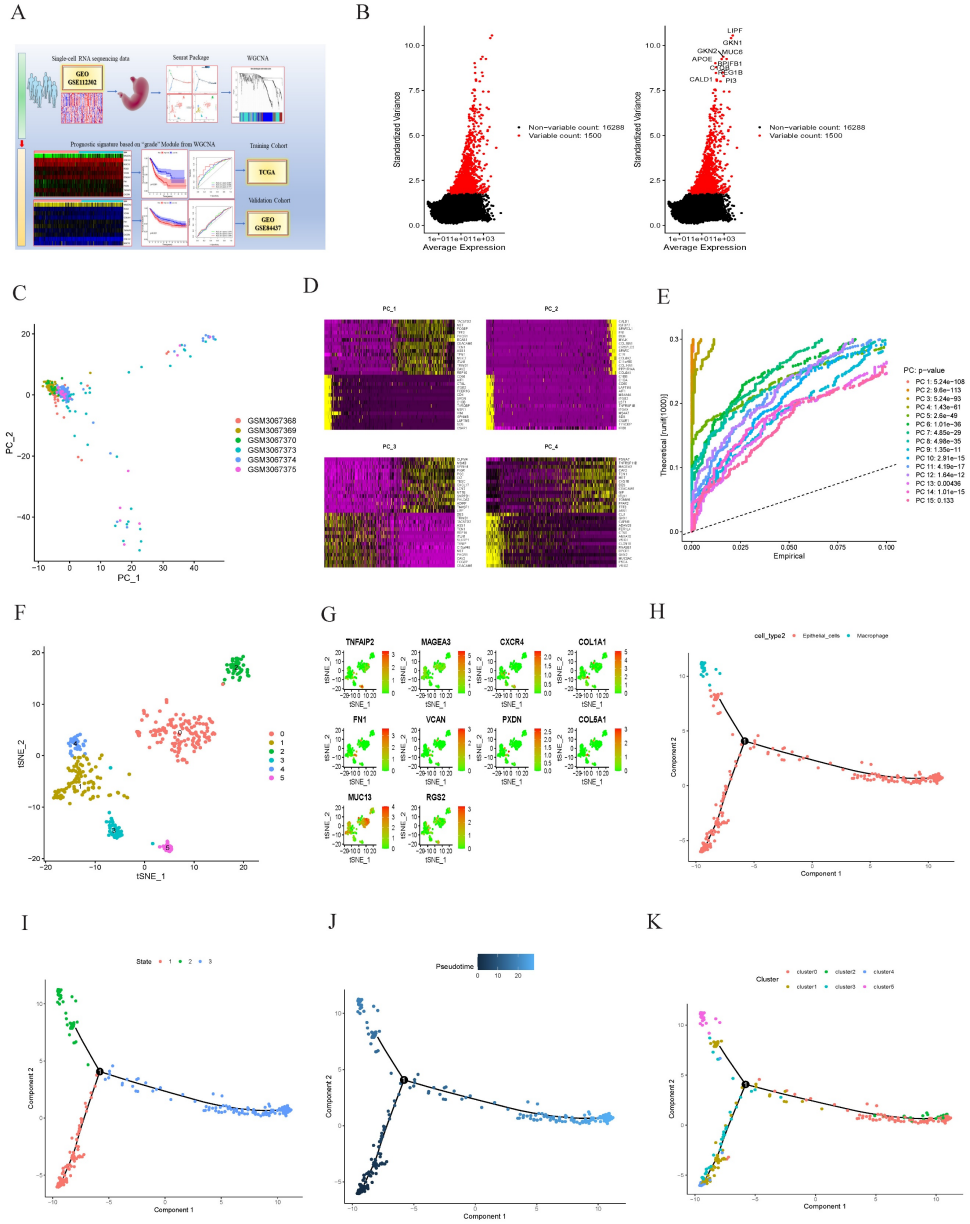
** Intercellular heterogeneity analysis in gastric cancer by scRNA-seq.** (A) The study of workflow based on single-cell RNA-seq in GC. (B) Variance diagram of the top ten differentially expressed genes identification in 1500 genes. (C), (D) and (E) The principal component analysis (PCA) showing the critical cell categories. (F) TSNE algorithm revealing the cells into six clusters. (G) The expression levels of 10 genes constructed prognostic signature in six clusters from the scRNA-seq. (H), (I), (J) and (K) cell annotations and trajectory analysis showed the tendency of cells differentiation.

The work flowchart is shown in Figure 1A, revealing the process of tumor differentiation grade-related genes prognostic signature construction based on scRNA-seq in GC.

### Patient tissues and clinical data collection

Our study was approved by The Ethical Committee of Tianjin Medical University General Hospital (IRB2024-YX-595-01). Gastric tissues (n=40) previously collected by us, along with clinical data, were used. All histopathology were performed for evaluated tumor differentiation grade of GC in patients from January 2021 to August 2022.

### Data processing

ScRNA-seq data from six primary gastric cancers and four normal gastric tissues were downloaded from GSE112302 dataset. Gene expression data was downloaded from GEO database including gastric cancer and normal tissues in GSE84437 for validation cohort. The original gene expression profiles and clinical data of GC in training cohort were obtained from the TCGA data portal. Firstly, the 'seurat' package was used to control data quality, and principal component analysis (PCA) was performed to conducted linear dimensionality reduction. Moreover, the t-Distributed Stochastic Neighbor Embedding (t-SNE) algorithm was applied to perform the cluster classification analysis and selected out the marker genes. Finally, the marker genes were annotated by the cluster and cell categories based on the 'SingleR' package (Version 0.99.13), and pseudotime analysis of cells was performed via the monocle package (Version 2.12.0).

### Functional enrichment analysis

R package 'ggplot2', 'enrichplot', 'clusterProfiler' and 'GOplot' was applied to perform Gene Ontology (GO) and Kyoto Encyclopedia of Genes and Genomes (KEGG) GO and KEGG enrichment analysis. Functional annotation with a P-value <0.05 was considered statistically significant. Gene Set Enrichment Analysis (GSEA) is utilized for exploring signaling pathways in GC between the low and the high tumor differentiation grade risk.

### The correlation of COL5A1 with tumor-infiltrating immune cells (TIICs)

CIBERSORT analysis tool is the deconvolution algorithm on basis of gene expression patterns for expressing tissue cell composition^14^. The 'CIBERSORT.R' package was performed to generate a proportion matrix of TIICs. The correlation of COL5A1 with TIICs was analyzed in this study.

### Development and validation of tumor differentiation grade-related genes prognostic signature

WGCNA analysis was used to identify the gene association patterns between different samples and highly coordinated gene sets^15^. The significant modules related to clinical traits were screened out using WGCNA. DEGs based on tumor differentiation grade module from WGCNA analysis were used to establish a multivariable Cox regression model. The risk score formula was constructed according to the previous formula^16^.

### Protein-protein interaction (PPI) and overall survival of tumor differentiation grade-related genes in GC

STRING database (https://cn.string-db.org/) was a functional protein association network, assembling all known and predicted proteins. The protein-protein interaction network of ten tumor differentiation grade-related genes was analyzed by STRING database. The Kaplan Meier plotter was applied to evaluate the correlation between the expression of all genes (mRNA, miRNA, protein) and survival in 30k+ samples from 21 tumor types^17^. The tool's primary purpose is the discovery and validation of survival biomarkers.

### H&E staining

Histopathological examinations were performed by two experienced pathologists in our hospital. Three to four HE stained levels/sections were examined per specimen.

### Statistical analysis

R software (version 4.0.0) was used to perform all statistical analyses in this study. Cox regression analysis, Kaplan-Meier curves with the log-rank test, receiver operating characteristic (ROC) curve the corresponding area under the ROC curve (AUC) were conducted by the 'glmnet', 'survival' and 'survivalROC' packages. Statistical significance was set at P < 0.05.

## Results

### ScRNA-seq profiling of the tumor landscape in primary GCs and normal gastric tissues

With scRNA-seq, 401 cell samples were acquired from 6 patient-derived GC tissues and 4 gastric normal tissues. Firstly, the study of quality control and data filtering was performed. Subsequently, the variance analysis revealed CALD1, PI3, REG1B, C1QB, APOE, BPIFB1, GKN2, MUC6, GKN1 and LIPF were the top ten significant DEGs in 1500 genes (Figure 1B). The PCA method was performed to screen out the significantly correlated genes in each component (Figure 1C-E). We also used the t-SNE algorithm successfully classified the samples into six clusters (Figure 1F). The expression of tumor differentiation grade-related genes prognostic signature in clusters (TNFAIP2, MAGEA3, CXCR4, COL1A1, FN1, VCAN, PXDN, COL5A1, MUC13 and RGS2) was shown in Fig.1G. Additionally, cell type was annotated for each cell sample, and all the cells in the six clusters were annotated as epithelial cells and macrophage (Figure 1H). Cell transition trajectory analyses showed that marker genes expression patterns changed in three ways, and we divided the cells into three branches: branch 1, branch 2, and branch 3. Branch 2 mainly represented macrophages. (Figure 1I-K).

### Functional enrichment analysis of common DEGs from cell transition trajectory

**Figure 2:**
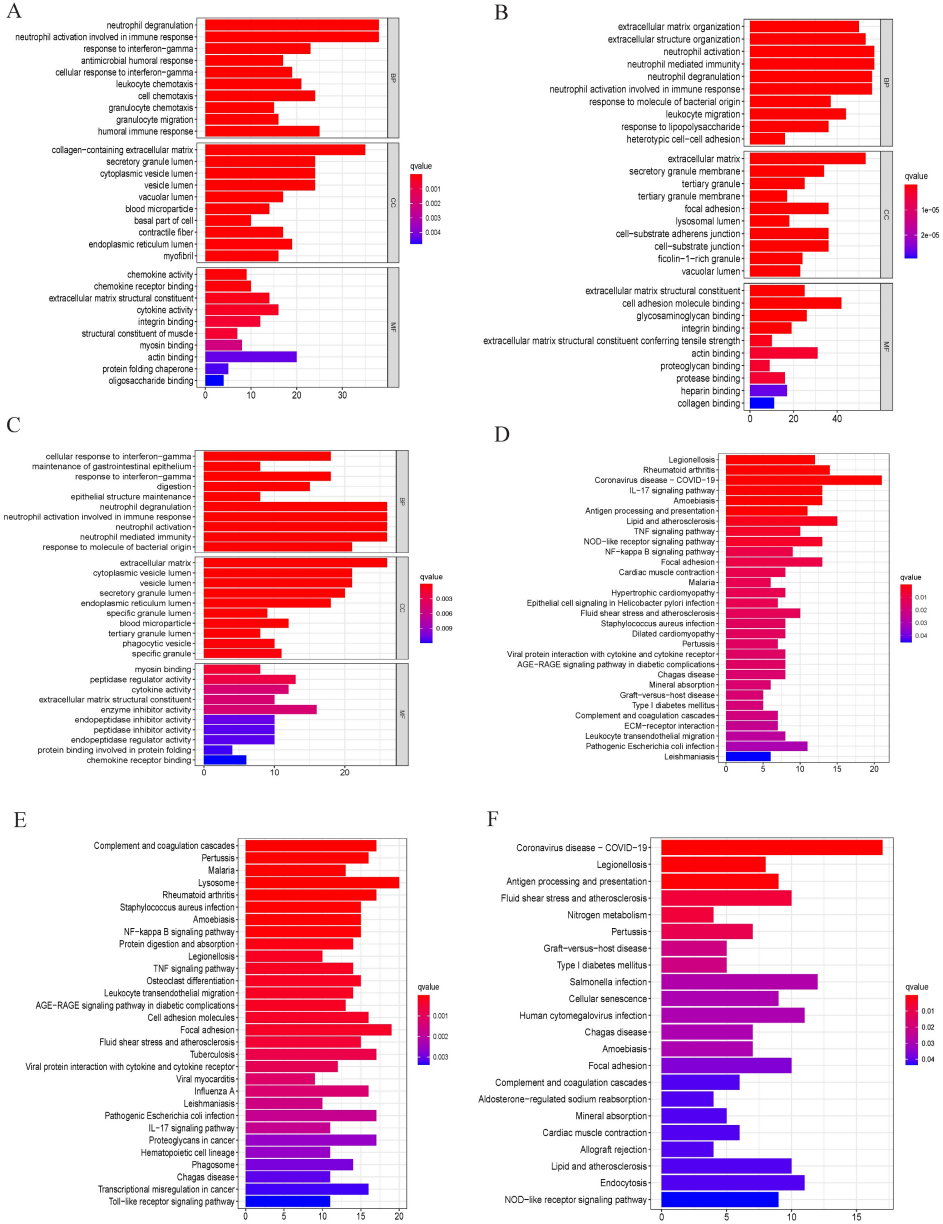
** GO and KEGG enrichment analysis.** (A), (B) and (C) GO analysis of common differentially expressed genes from branch 1, branch 2 and branch 3. “BP” represents “biological process,” “CC” represents “cellular component” and “MF” represents “molecular function.” (D), (E) and (F) Kyoto Encyclopedia of Genes and Genomes analysis of differentially expressed genes from branch 1, branch 2 and branch 3.

To investigate the biological functional of common DEGs based on branch 1, branch 2 and branch 3, GO and KEGG were performed by R package 'ggplot2', 'enrichplot', 'clusterProfiler' and 'GOplot'. In this study, we found that neutrophil degranulation, neutrophil activation involved in immune response, neutrophil mediated immunity and neutrophil activation played an important role in biological processes (BP), cell components (CC), and molecular functions (MF) of GO terms (Figure 2A-C). In addition, we observed that common DEGs from cell transition trajectory were mainly enriched in coronavirus disease- COVID-19, legionellosis, rheumatoid arthritis, complement and coagulation cascades, pertussis, malaria, lysosome, antigen processing and presentation (Figure 2D-F). Above results showed that neutrophil could play an important role in GC.

### Tumor differentiation grade-related genes identification

**Figure 3:**
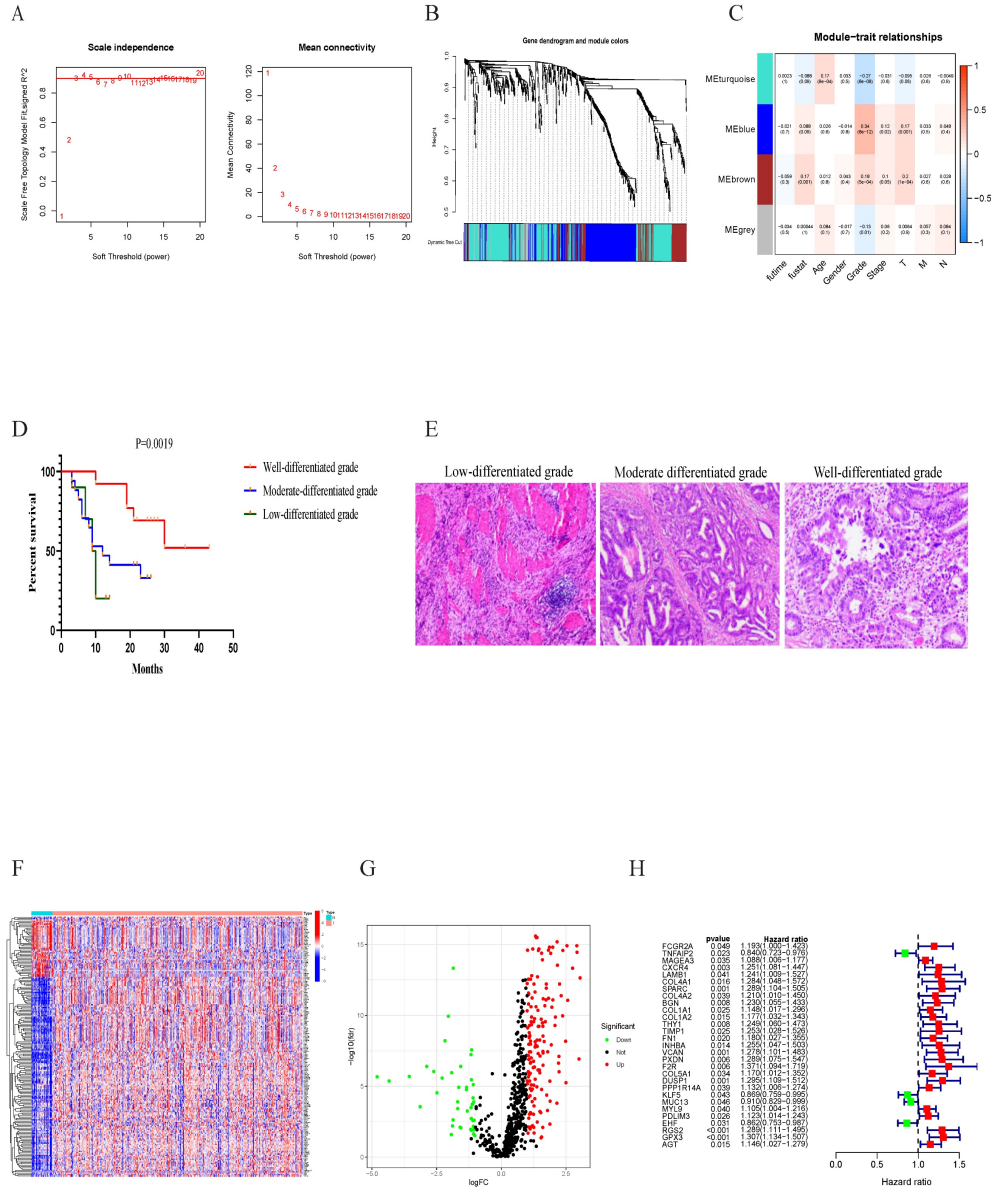
** Tumor differentiation grade module based on grade identification.** (A) and (B) determination of soft-threshold power in the WGCNA. (C) Heatmap displaying the relationship of the module with clinical traits. The MEblue-, MEgrey- MEbrown- MEturquoise-grade modules were screened out for further analysis. (D) Kaplan-Meier analysis revealed that well differentiated grade had better prognosis in 40 GC patients. (E) H&E staining identified three group: well differentiated grade, moderate-differentiated grade, and low-differentiated grade. (F) Heatmap and (G) volcano plot of differentially expressed genes in MEblue-, MEgrey- MEbrown- MEturquoise-grade modules. (H) Forest map of 29 prognostic genes related with grade module by univariate Cox regression.

After data preprocessing, a total of 618 common DEGs from three branches were used to perform weighted gene co-expression network analysis (WGCNA). We also observed that 618 genes were also common in the expression matrix of GSE84437 and TCGA. The adjacency matrix and the topological overlap matrix were constructed (Figure 3A). Next, four modules were identified based on average hierarchical clustering and dynamic tree clipping (Figure 3B). Histological grading system revealed the extent of malignancy of GC. Jayanthi, V. et al. constructed grade-specific molecular interaction networks identified grade-specific biomarkers for breast cancer^18^. Then, we found that tumor differentiation grade was significantly associated with prognosis in GC. In this study, the blue module, brown module, grey module and turquoise module were highly related to tumor differentiation grade. Four modules were selected as a clinically important module for further analysis (Figure 3C). Kaplan-Meier analysis revealed that well differentiated grade had better prognosis in 40 GC patients (Figure 3D). H&E staining identified three group: well differentiated grade, moderate-differentiated grade, and low-differentiated grade (Figure 3E). Finally, a total of 190 differentially expressed genes were identified, as shown in Figure 3F, 3G.

We then integrated the clinical data from all patients of TCGA dataset, univariate analysis revealed that the expression of FCGR2A, TNFAIP2, MAGEA3, CXCR4, LAMB1, COL4A1, SPARC, COL4A2, BGN, COL1A1, COL1A2, THY1, TIMP1, FN1, INHBA, VCAN, PXDN, F2R, COL5A1, DUSP1, PPP1R14A, KLF5, MUC13, MYL9, PDLIM3, EHF, RGS2, GPX3, and AGT were distinctly associated with prognosis in GC patients (Figure 3H).

### Risk model construction of tumor differentiation grade-related genes and external data validation

**Figure 4:**
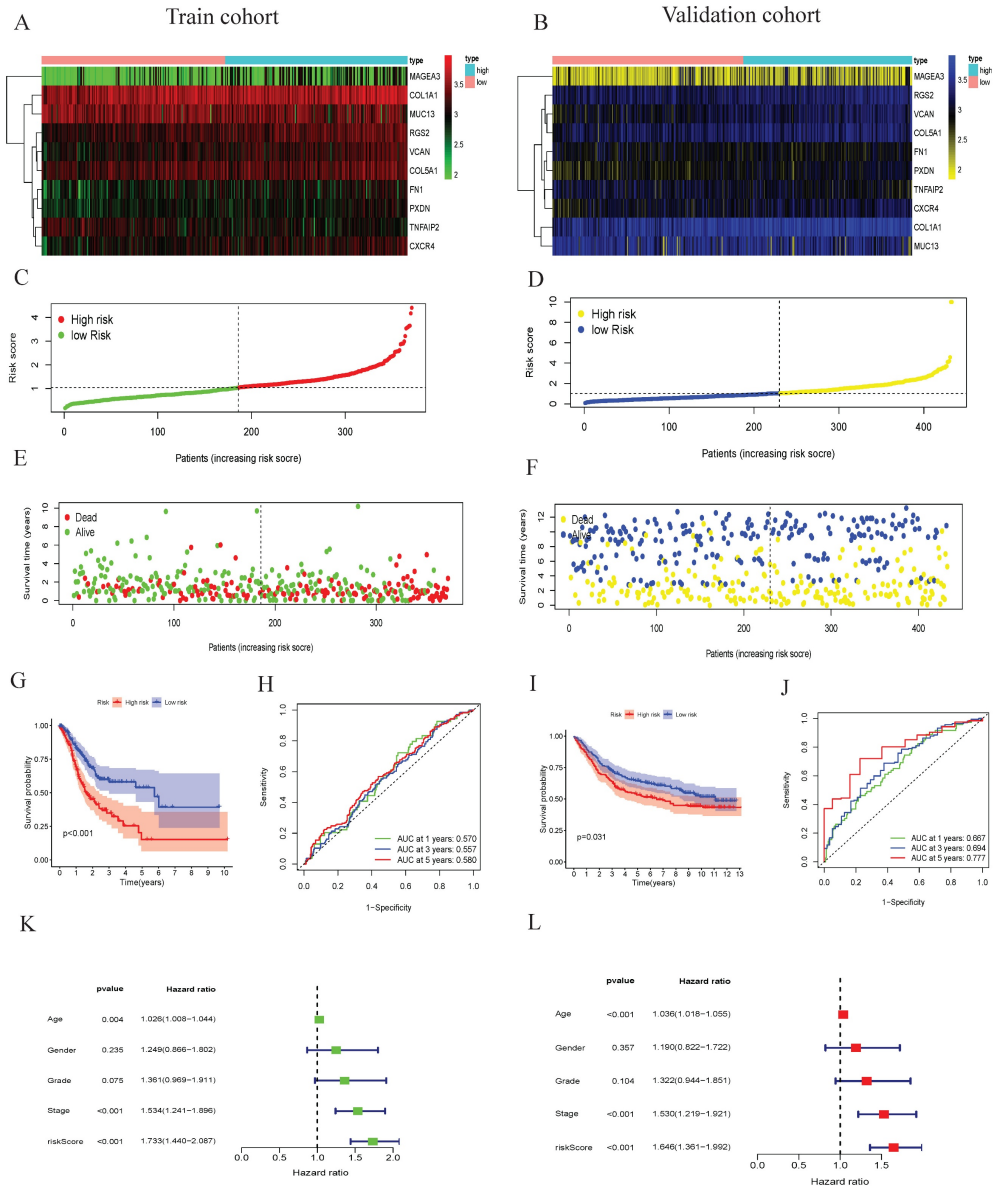
** The tumor differentiation grade-related genes prognostic signature construction and external validation.** Heatmap of gene expression pattern (A), the risk scores distribution (C), survival status of every patient (E), the Kaplan-Meier plot showing OS of patients in high and low risk groups (G), time-dependent ROC curves for the prognostic signature based on grade module (H) in training cohort. Heatmap of gene expression pattern (B), the risk scores distribution (D), survival status of patients (F), the Kaplan-Meier curve in different risk groups (I), time-dependent ROC curves for the prognostic signature based on grade module (J) in validation cohort. Forest plot of associations between risk factors and the survival of gastric cancer in (K)Univariate and (L)Multivariate COX regression analysis.

We then selected ten genes based on the coefficients derived from the multivariate analysis to construct risk model. The coefficients of the ten prognostic genes are shown in Table 1. The study formula for the risk score was as follows^19^: Risk score = ( -0.209406939 × expression value of TNFAIP2) + ( 0.126113147 × expression value of MAGEA3) + ( 0.171457823 × expression value of CXCR4) + ( 0.58121411 × expression value of COL1A1)+( -0.2694724× expression value of FN1 ) + ( 0.345428991 × expression value of VCAN)+( -0.800354177 × expression value of COL5A1)+( -0.098651917 × expression value of MUC13) + ( 0.345428991 × expression value of PXDN) + ( 0.202535842 × expression value of RGS2). To investigate genes expression profiles in high-risk and low-risk GC groups, gene heat map, the risk score distribution and follow-up time were shown in Figure 4A,4C,4E. The Kaplan-Meier plot revealed that patients in the high-risk group had a significantly poorer OS than those in the low-risk group in the train cohort (Figure 4G). The prognostic signature had a good accuracy to predict OS in the train cohort with 1-year, 3-year and 5-year AUC of 0.570, 0.557, 0.580 (Figure 4H).

The prognostic signature associated with grade module was validated using the same risk score formula and the same cut-off value. The gene expression pattern, risk distribution, survival status, the Kaplan-Meier plot and AUC were shown in Figure 4B, 4D, 4F, 4I, 4J. Multivariate Cox regression analyses showed that age, stage and the risk score were found to be independent risk factors for prognosis in patients with gastric cancer (Figure 4K-L). These analyses indicated that tumor differentiation grade-related genes and constructed risk prognostic models have good prognostic value.

### GSEA enrichment and Kaplan-Meier Plotter analysis

**Figure 5:**
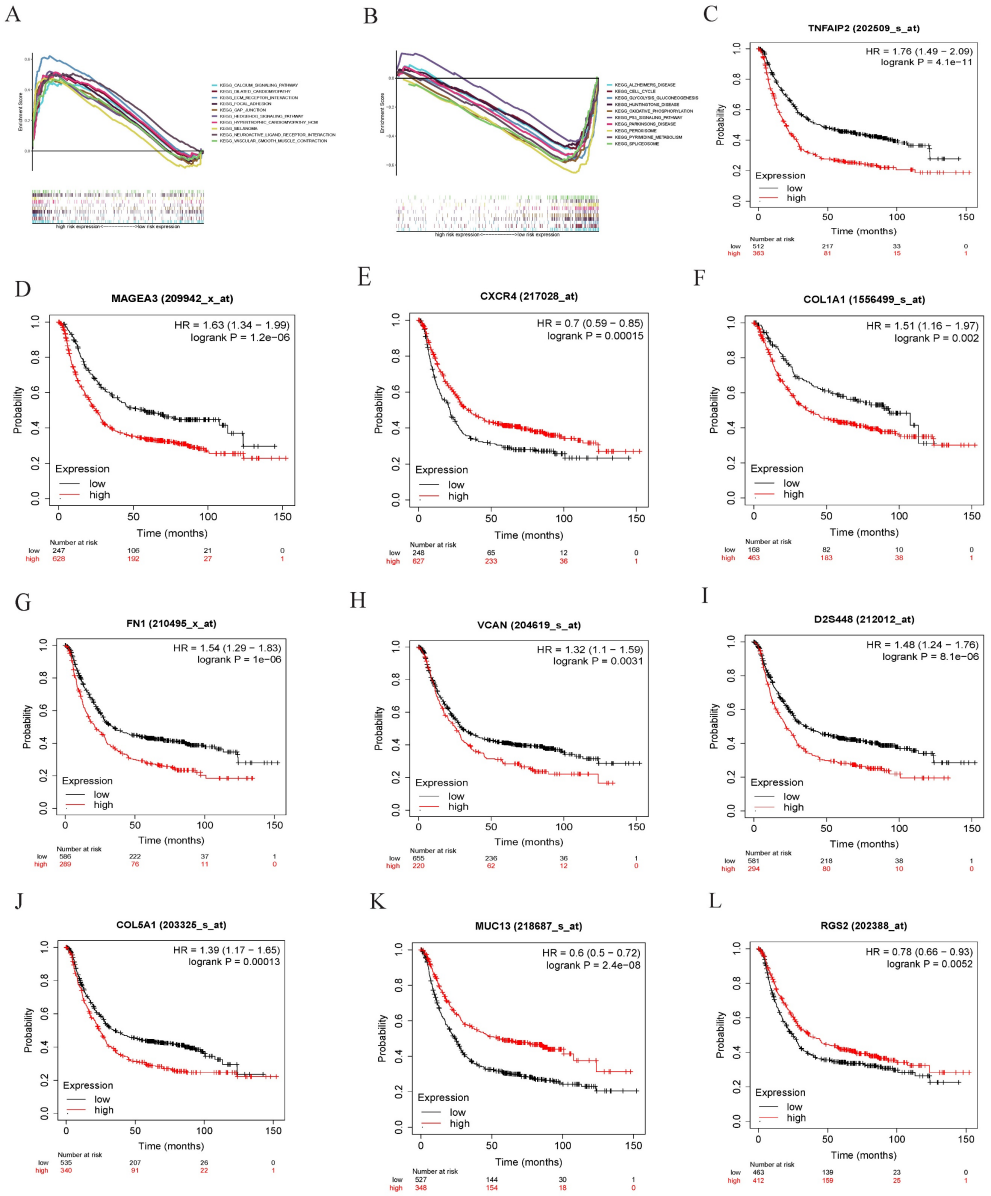
**GSEA enrichment analysis and overall survival in GC.** (A) The top ten functional enrichment pathways of grade-related prognostic signature in high-risk groups, (B) The top ten functional enrichment pathways of grade-related prognostic signature in low-risk groups. Overall survival analysis revealed that the expression of the tumor differentiation grade-related genes (C) NFAIP2, (D) MAGEA3, (E) CXCR4, (F) COL1A1, (G) FN1, (H) VCAN, (I) PXDN, (J) COL5A1, (K) MUC13 and (L) RGS2 was obviously associated with prognosis in GC.

In this study, GSEA software were applied to analyze the low-risk group and the high-risk group of tumor differentiation grade-related genes signature. We showed that grade-related genes in this signature are significantly enriched in the high-risk (Figure 5A) and low-risk groups (Figure 5B). The top five KEGG pathways in the high-risk group were neuroactive ligand receptor interaction, ECM receptor interaction, GAP junction, hypertrophic cardiomyopathy HCM, and focal adhesion. However, the top five KEGG pathways in the low-risk group were peroxisome, pyrimidine metabolism, glycolysis gluconeogenesis, alzheimers disease, and oxidative phosphorylation. Kaplan-Meier survival analysis suggested that the expression of TNFAIP2, MAGEA3, CXCR4, COL1A1, FN1, VCAN, PXDN, COL5A1, MUC13, and RGS2 had significantly worse prognosis (P < 0.05) (Figure 5C-L).

### COL5A1 was significant differentially expressed and associated with immune cells

**Figure 6:**
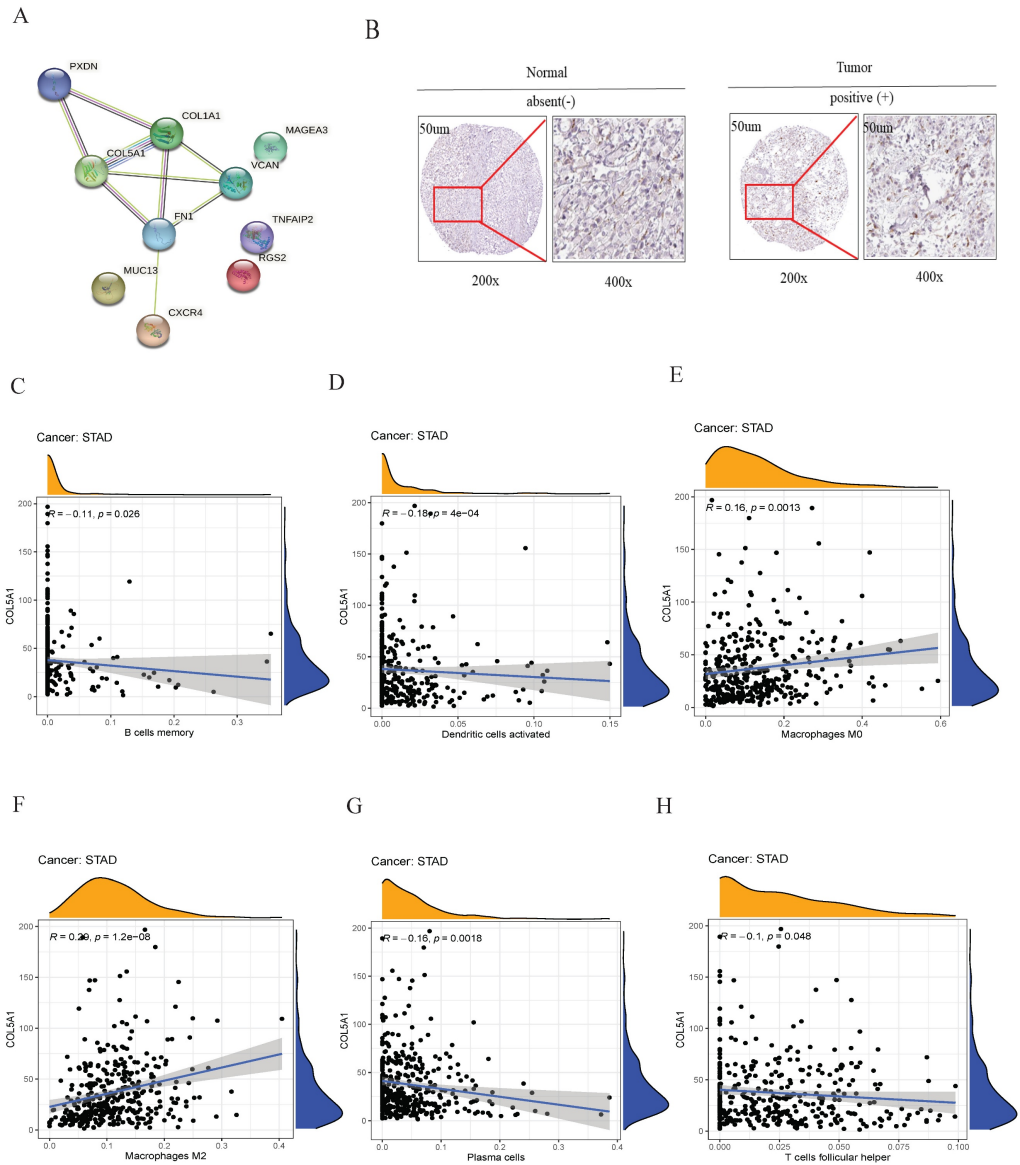
**PPI and the relationship of COL5A1 with immune cell infiltrating.** (A) the interaction among ten tumor differentiation grade-related genes, (B) IHC revealed that COL5A1 was upregulated in GC, (C) B cells memory, (D) dendritic cells activated, (E) macrophages M0, (F) macrophages M2, (G) plasma cells, and (H) T cells follicular helper were significantly associated with COL5A1 expression in GC.

Among tumor differentiation grade-related genes in the prognostic signature, collagen type V alpha 1 (COL5A1) is a key molecular node (Figure 6A). Therefore, COL5A1, a risk factor of the risk model, was analyzed for subsequently experimental verification. We found that COL5A1 was highly expressed in the GC tissues compared to the adjacent normal tissues in the Human Protein Atalas webserver (Figure 6B). Subsequently, we observed that the expression of COL5A1 was significantly associated with B cells memory (Figure 6C), dendritic cells activated (Figure 6D), macrophages M0 (Figure 6E), macrophages M2 (Figure 6F), plasma cells (Figure 6G), T cells follicular helper (Figure 6H). These data showed that COL5A1 could play an important role in tumor microenvironment in GC.

## Discussion

Morphological heterogeneity and genetic heterogeneity comprise tumor heterogeneity affecting diagnosis and therapy in cancer^20^. Gastric cancer is a highly heterogeneous malignant cancer with virous subtypes and clinical behaviors^21^, ^22^. Here, we explored tumor heterogeneity at the single cell level in GC using scRNA-seq.

Our result based on scRNA-seq analysis showed that tumors included virous cells, such as malignant cells, tumor infiltrating cells and stromal components. Cell transition trajectory analysis is widely used to explore different cell types at different stages of development and differentiation in several reports^23^, ^24^. We identified three branches in cell transition trajectory. In this study, common DEGs were analyzed in three branches. Our analysis revealed that these genes were enriched in Coronavirus disease 2019 (COVID-19). COVID-19 is defined as a respiratory tract infection caused by the severe acute respiratory syndrome (SARS) coronavirus (COV), also named SARS-CoV-2^25^. COVID-19 was first found in Central China (Wuhan, the capital of Hubei province) at the end of December 2019^26^. Many reports have explored the relationship of COVID-19 with cancer^27^-^29^. Hoang, T. et al reported that genetic susceptibility of ACE2 and TMPRSS2 in GC was associated with the susceptibility to COVID-19 infection^30^.

The tumor microenvironment (TME) consists of a heterogenous cellular component affecting cancer cell behavior. Sathe, A. et al showed that gastric cancer TME was significantly enriched for stromal cells, macrophages, dendritic cells (DC), and Tregs^31^. Our study also found that cells cluster 5 was main macrophages. We investigated that common DEGs in three branches were associated with neutrophil degranulation, neutrophil activation involved in immune response, neutrophil mediated immunity and neutrophil activation. Increasing studies revealed that neutrophil played a critical role in tumor microenvironment^32^-^34^.

In this study, we used ten tumor differentiation grade-related genes for the first time to establish the prognostic signature including TNFAIP2, MAGEA3, CXCR4, COL1A1, FN1, VCAN, PXDN, COL5A1, MUC13, and RGS2 based on single-cell RNA-seq. More importantly, age, stage and riskscore were independent prognostic factors. Using H&E, we evaluated tumor differentiation and found that patients in well differentiated grade group had better prognosis than other groups. To our knowledge, this is the first study that directly revealed that a prognostic risk model based on tumor differentiation grade-related genes could predict prognosis in GC.

Many recent studies showed that TNFAIP2, MAGEA3, CXCR4, COL1A1, FN1, VCAN, PXDN, COL5A1, MUC13, and RGS2 could predict overall survival as a single gene biomarker^35^-^43^. Here, we found that the expression level of TNFAIP2, MAGEA3, CXCR4, COL1A1, FN1, VCAN, PXDN, COL5A1, MUC13, and RGS2 is closely related to the prognosis of GC patients.

Several studies have used PPI network to study the interactome of protein and screen hub genes^44^. Our PPI network studies demonstrated that COL5A1 was a key molecular node. As the collagen family members, high level of COL5A1 is closely associated with the poor prognosis of multiple human tumors^45^, ^46^. Focused on the molecular role of COL5A1 in malignant cells, investigators have reported that NAT10 promoted GC metastasis via N4-acetylated COL5A1^47^. We also observed that COL5A1 was differentially expressed in GC tissues. A previous study shows that COL5A1 was a cancer-associated fibroblast gene signature as a poor prognostic factor and potential therapeutic target in GC^48^. Given that the correlations of COL5A1 with immune infiltrating cells, our results revealed that COL5A1 was significantly correlated with B cell memory, dendritic cells-activated, macrophage M0, macrophage M2, plasma cells, and T cells follicular helper, suggesting its role in regulating TME. However, this study had no enough clinical samples to validate this prognostic signature using our experimental data.

## Conclusions

Together, we screened tumor differentiation grade-related genes prognostic signature including NFAIP2, MAGEA3, CXCR4, COL1A1, FN1, VCAN, PXDN, COL5A1, MUC13 and RGS2, and provided evidence of GC heterogeneity based on single-cell RNA-seq. Importantly, COL5A1 may be as a novel therapeutic target and biomarkers for GC.

## Figures and Tables

**Table 1:** The coefficients of the ten prognostic genes based on scRNA-seq in GC

id	coef	HR	HR.95L	HR.95H	pvalue
TNFAIP2	-0.209	0.811	0.681	0.966	0.019
MAGEA3	0.126	1.134	1.043	1.234	0.003
CXCR4	0.171	1.187	0.993	1.419	0.060
COL1A1	0.581	1.788	1.160	2.758	0.009
FN1	-0.269	0.764	0.581	1.004	0.054
VCAN	0.267	1.306	0.941	1.814	0.110
PXDN	0.345	1.413	1.007	1.981	0.045
COL5A1	-0.800	0.449	0.241	0.838	0.012
MUC13	-0.099	0.906	0.813	1.010	0.075

## Abbreviations

GC: gastric cancer
scRNA-seq: single-cell RNA sequencing
WGCNA: weighted gene co-expression network analysis
H&E: hematoxylin and eosin
DEGs: differentially expressed genes
PPI: protein-protein interaction
PCA: principal component analysis
GO: gene ontology
KEGG: kyoto encyclopedia of genes and genomes
GSEA: gene set enrichment analysis
ROC: receiver operating characteristic
AUC: area under curve
TIICs: tumor-infiltrating immune cells
t-SNE: t-distributed stochastic neighbor embedding
BP: biological processes
CC: cell components
MF: molecular functions
TME: tumor microenvironment
DC: dendritic cells
COL5A1: collagen type V alpha 1

## References

[1] Sung H, Ferlay J, Siegel RL, Laversanne M, Soerjomataram I, Jemal A (2021). Global cancer statistics 2020: GLOBOCAN estimates of incidence and mortality worldwide for 36 cancers in 185 countries. CA Cancer J Clin.

[2] Ahn SH, Kang SH, Lee Y, Min SH, Park YS, Park DJ (2019). Long-term Survival Outcomes of Laparoscopic Gastrectomy for Advanced Gastric Cancer: Five-year Results of a Phase II Prospective Clinical Trial. Journal of gastric cancer.

[3] Digklia A, Wagner AD (2016). Advanced gastric cancer: Current treatment landscape and future perspectives. World J Gastroenterol.

[4] Ghafouri-Fard S, Vafaee R, Shoorei H, Taheri M (2020). MicroRNAs in gastric cancer: Biomarkers and therapeutic targets. Gene.

[5] Ouyang J, Long Z, Li G (2020). Circular RNAs in Gastric Cancer: Potential Biomarkers and Therapeutic Targets. Biomed Res Int.

[6] Rojas A, Araya P, Gonzalez I, Morales E (2020). Gastric Tumor Microenvironment. Adv Exp Med Biol.

[7] Derwinger K, Kodeda K, Bexe-Lindskog E, Taflin H (2010). Tumour differentiation grade is associated with TNM staging and the risk of node metastasis in colorectal cancer. Acta Oncol.

[8] Kalogeraki A, Tzardi M, Zoras O, Giannikaki E, Papadakis M, Tamiolakis D (2010). Apoptosis and cell proliferation correlated with tumor grade in patients with lung adenocarcinoma. In vivo.

[9] Fu K, Hui B, Wang Q, Lu C, Shi W, Zhang Z (2020). Single-cell RNA sequencing of immune cells in gastric cancer patients. Aging.

[10] Mao X, Yang X, Chen X, Yu S, Yu S, Zhang B (2021). Single-cell transcriptome analysis revealed the heterogeneity and microenvironment of gastrointestinal stromal tumors. Cancer Sci.

[11] Sathe A, Grimes SM, Lau BT, Chen J, Suarez C, Huang RJ (2020). Single-Cell Genomic Characterization Reveals the Cellular Reprogramming of the Gastric Tumor Microenvironment. Clin Cancer Res.

[12] Zhang J, Guan M, Wang Q, Zhang J, Zhou T, Sun X Single-cell transcriptome-based multilayer network biomarker for predicting prognosis and therapeutic response of gliomas. 2020; 21: 1080-97.

[13] Kim KT, Lee HW, Lee HO, Kim SC, Seo YJ, Chung W (2015). Single-cell mRNA sequencing identifies subclonal heterogeneity in anti-cancer drug responses of lung adenocarcinoma cells. Genome Biol.

[14] Newman AM, Liu CL, Green MR, Gentles AJ, Feng W, Xu Y (2015). Robust enumeration of cell subsets from tissue expression profiles. Nat Methods.

[15] Langfelder P, Horvath S (2008). WGCNA: an R package for weighted correlation network analysis. BMC Bioinformatics.

[16] Fu D, Zhang B, Yang L, Huang S, Xin W (2020). Development of an Immune-Related Risk Signature for Predicting Prognosis in Lung Squamous Cell Carcinoma. Front Genet.

[17] Nagy A, Lanczky A, Menyhart O, Gyorffy B (2018). Author Correction: Validation of miRNA prognostic power in hepatocellular carcinoma using expression data of independent datasets. Sci Rep.

[18] Jayanthi V, Das AB, Saxena U (2020). Grade-specific diagnostic and prognostic biomarkers in breast cancer. Genomics.

[19] Wang Z, Wang Z, Niu X, Liu J, Wang Z, Chen L (2019). Identification of seven-gene signature for prediction of lung squamous cell carcinoma. Onco Targets Ther.

[20] Zhang P, Yang M, Zhang Y, Xiao S, Lai X, Tan A (2019). Dissecting the Single-Cell Transcriptome Network Underlying Gastric Premalignant Lesions and Early Gastric Cancer. Cell Rep.

[21] Zhuang Y, Peng LS, Zhao YL, Shi Y, Mao XH, Guo G Increased intratumoral IL-22-producing CD4(+) T cells and Th22 cells correlate with gastric cancer progression and predict poor patient survival. 2012; 61: 1965-75.

[22] Zhou Q, Wu X, Wang X, Yu Z, Pan T, Li Z (2020). The reciprocal interaction between tumor cells and activated fibroblasts mediated by TNF-α/IL-33/ST2L signaling promotes gastric cancer metastasis. Oncogene.

[23] Chu LF, Leng N, Zhang J, Hou Z, Mamott D, Vereide DT (2016). Single-cell RNA-seq reveals novel regulators of human embryonic stem cell differentiation to definitive endoderm. Genome Biol.

[24] Zhou Y, Liu Z, Welch JD, Gao X, Wang L, Garbutt T Single-Cell Transcriptomic Analyses of Cell Fate Transitions during Human Cardiac Reprogramming. 2019; 25: 149-64.e9.

[25] Passaro A, Peters S, Mok TSK, Attili I, Mitsudomi T, de Marinis F (2020). Testing for COVID-19 in lung cancer patients. Ann Oncol.

[26] Tian S, Hu W, Niu L, Liu H, Xu H, Xiao SY (2020). Pulmonary Pathology of Early-Phase 2019 Novel Coronavirus (COVID-19) Pneumonia in Two Patients With Lung Cancer. J Thorac Oncol.

[27] Monin L, Laing AG, Muñoz-Ruiz M, McKenzie DR, Del Molino Del Barrio I, Alaguthurai T (2021). Safety and immunogenicity of one versus two doses of the COVID-19 vaccine BNT162b2 for patients with cancer: interim analysis of a prospective observational study. Lancet Oncol.

[28] Griffiths EA, Segal BH (2021). Immune responses to COVID-19 vaccines in patients with cancer: Promising results and a note of caution. Cancer Cell.

[29] Potter DA, Thomas A, Rugo HS (2021). A Neoadjuvant Chemotherapy Trial for Early Breast Cancer is Impacted by COVID-19: Addressing Vaccination and Cancer Trials Through Education, Equity, and Outcomes. Clin Cancer Res.

[30] Hoang T, Nguyen TQ, Tran TTA (2021). Genetic Susceptibility of ACE2 and TMPRSS2 in Six Common Cancers and Possible Impacts on COVID-19. Cancer Res Treat.

[31] Sathe A, Grimes SM, Lau BT, Chen J, Suarez C, Huang RJ (2020). Single cell genomic characterization reveals the cellular reprogramming of the gastric tumor microenvironment. Clin Cancer Res.

[32] Lee W, Ko SY, Mohamed MS, Kenny HA, Lengyel E, Naora H (2019). Neutrophils facilitate ovarian cancer premetastatic niche formation in the omentum. J Exp Med.

[33] Giese MA, Hind LE, Huttenlocher A (2019). Neutrophil plasticity in the tumor microenvironment. Blood.

[34] Ocana A, Nieto-Jiménez C, Pandiella A, Templeton AJ (2017). Neutrophils in cancer: prognostic role and therapeutic strategies. Mol Cancer.

[35] Niwa N, Tanaka N, Hongo H, Miyazaki Y, Takamatsu K, Mizuno R (2019). TNFAIP2 expression induces epithelial-to-mesenchymal transition and confers platinum resistance in urothelial cancer cells. Lab Invest.

[36] Craig AJ, Garcia-Lezana T, Ruiz de Galarreta M, Villacorta-Martin C, Kozlova EG, Martins-Filho SN (2021). Transcriptomic characterization of cancer-testis antigens identifies MAGEA3 as a driver of tumor progression in hepatocellular carcinoma. PLoS Genet.

[37] Lecavalier-Barsoum M, Chaudary N, Han K, Koritzinsky M, Hill R, Milosevic M (2018). Targeting the CXCL12/CXCR4 pathway and myeloid cells to improve radiation treatment of locally advanced cervical cancer. Int J Cancer.

[38] Verginadis II, Avgousti H Monslow J, Skoufos G Chinga F, Kim K et al (2022). A stromal Integrated Stress Response activates perivascular cancer-associated fibroblasts to drive angiogenesis and tumour progression. Nat Cell Biol.

[39] Glasner A, Levi A, Enk J, Isaacson B, Viukov S, Orlanski S (2018). NKp46 Receptor-Mediated Interferon-γ Production by Natural Killer Cells Increases Fibronectin 1 to Alter Tumor Architecture and Control Metastasis. Immunity.

[40] Mafu TS, September AV, Shamley D (2021). Regulatory VCAN polymorphism is associated with shoulder pain and disability in breast cancer survivors. Hum Genomics.

[41] Dempsey B, Cruz LC, Mineiro MF, da Silva RP, Meotti FC (2022). Uric Acid Reacts with Peroxidasin, Decreases Collagen IV Crosslink, Impairs Human Endothelial Cell Migration and Adhesion. Antioxidants (Basel, Switzerland).

[42] Gu S, Peng Z, Wu Y, Wang Y, Lei D, Jiang X (2021). COL5A1 Serves as a Biomarker of Tumor Progression and Poor Prognosis and May Be a Potential Therapeutic Target in Gliomas. Frontiers in oncology.

[43] Tiemin P, Fanzheng M, Peng X, Jihua H, Ruipeng S, Yaliang L (2020). MUC13 promotes intrahepatic cholangiocarcinoma progression via EGFR/PI3K/AKT pathways. J Hepatol.

[44] Wimalagunasekara SS, Weeraman J, Tirimanne S, Fernando PC (2023). Protein-protein interaction (PPI) network analysis reveals important hub proteins and sub-network modules for root development in rice (Oryza sativa). Journal, genetic engineering & biotechnology.

[45] Li P, Cao J, Li J, Yao Z, Han D, Ying L (2020). Identification of prognostic biomarkers associated with stromal cell infiltration in muscle-invasive bladder cancer by bioinformatics analyses. Cancer Med.

[46] Zhu H, Hu X, Feng S, Jian Z, Xu X, Gu L (2022). The Hypoxia-Related Gene COL5A1 Is a Prognostic and Immunological Biomarker for Multiple Human Tumors. Oxid Med Cell Longev.

[47] Zhang Y, Jing Y, Wang Y, Tang J, Zhu X, Jin WL (2021). NAT10 promotes gastric cancer metastasis via N4-acetylated COL5A1. Signal transduction and targeted therapy.

[48] Ucaryilmaz Metin C, Ozcan G (2022). Comprehensive bioinformatic analysis reveals a cancer-associated fibroblast gene signature as a poor prognostic factor and potential therapeutic target in gastric cancer. BMC Cancer.

